# Exploring the potential of cold plasma treatment followed by zinc-priming for biofortification of buckwheat sprouts

**DOI:** 10.3389/fnut.2023.1151101

**Published:** 2023-05-05

**Authors:** Pia Starič, Lucija Remic, Katarina Vogel-Mikuš, Ita Junkar, Primož Vavpetič, Mitja Kelemen, Paula Pongrac

**Affiliations:** ^1^Jožef Stefan Institute, Ljubljana, Slovenia; ^2^Biotechnical Faculty, University of Ljubljana, Ljubljana, Slovenia

**Keywords:** common buckwheat, *Fagopyrum esculentum*, non-thermal plasma, zinc distribution, grain tissues

## Abstract

Increasing the concentration of an element in edible produce (i.e., biofortification) can mitigate the element deficiency in humans. Sprouts are small but popular part of healthy diets providing vitamins and essential elements throughout the year. Element composition of sprouts can easily be amended, e.g., by soaking the grains in element-rich solution before germination (grain-priming). In addition, pre-treatment of grains to improve element translocation from the solution into the grain may further enhance the element concentration in the sprout. Cold plasma technique could provide such solution, as it increases wettability and water uptake of grains. Grains of common buckwheat (*Fogopyrum esculentum* Moench) were pre-treated/ untreated with cold plasma and soaked in ZnCl_2_ solution/pure water. Germination tests, α-amylase activity, grain hydrophilic properties and water uptake were assessed. Element composition of grain tissues and of sprouts was assessed by micro-particle-induced-X-ray emission and X-ray fluorescence spectroscopy, respectively. Grain-priming increased Zn concentration in shoots of common buckwheat sprouts more than five-times, namely from 79 to 423 mg Zn kg^−1^ dry weight. Cold plasma treatment increased grain wettability and water uptake into the grain. However, cold plasma pre-treatment followed by grain-priming with ZnCl_2_ did not increase Zn concentration in different grain tissues or in the sprouts more than the priming alone, but rather decreased the Zn concentration in sprout shoots (average ± standard error: 216 ± 6.13 and 174 ± 7.57 mg Zn kg^−1^ dry weight, respectively). When the fresh weight portion of whole sprouts (i.e., of roots and shoots) was considered, comparable average requirements of Zn, namely 24.5 % and 35 % for adult men and women would be satisfied by consuming cold plasma pre-treated and not pre-treated grains. Potential advantages of cold plasma pre-treatment need to be tested further, mainly to optimize the duration of soaking required to produce Zn-enriched sprouts.

## 1. Introduction

Optimal development of organisms depends upon provision of sufficient amounts of essential elements. Since plants represent the base of the food chain and deliver between 83 and 94% of the essential elements to human diets (1) the variation in the element compositions of crops is of considerable importance for human nutrition (2). Seven elements (iron (Fe), zinc (Zn), magnesium (Mg), copper (Cu), calcium (Ca), selenium (Se) and iodine (I)) are often lacking in human diets, resulting in negative impact on health and wellbeing of more than two billion people worldwide (3, 4). Substantial progress to reduce malnutrition has been made in increasing concentrations of these elements in edible plant tissues by a process called biofortification. The increase in density of elements in a crop can be achieved through agronomic practices designed to increase phytoavailability of the deficient element by soil fertilization or by foliar application of element-containing compounds, as well as by plant breeding and/or transgenic techniques to develop plants with better element-use efficiency. When biofortified product is consumed regularly, measurable improvements in human health and nutrition can be observed (5).

Zinc deficiency compromises the immune system, increases the risk of arteriosclerosis, anemia and gastrointestinal disorders (6–8). There has been a considerable success in biofortifying crops with Zn, mostly achieved through soil fertilization and/or foliar application of Zn-rich compounds with species-specific degree of increase (9). An alternative to field-based fertilization, the soaking of seeds or grains in Zn solutions, known as seed/ grain-priming, has also been explored for improving Zn nutrition of the growing seedling (10–13), or, more recently, to grow Zn-rich edible sprouts. A significant increase in Zn concentration in shoots or/ and leaves of chickpeas, wheat or mung beans, primed in different concentrations of ZnSO_4_ has been reported (14, 15). Similarly, soaking grains in ZnCl_2_ resulted in increased concentration of Zn in above-ground plant tissues of wheat and peanut (16, 17). Up to five-fold increase in Zn concentration in peanut sprouts grown from primed seeds has been reported (17). It seems Zn seed/grain-priming could be a feasible approach to increase Zn intake, if sufficient amount of the Zn-biofortified product is consumed and the Zn present in product is bioavailable.

The rate of uptake of ions from the solution used to soak the seeds or grains depends on the surface area of the grain, the surface properties, the concentration of ions in the solution, the temperature of the solution and the soaking time. Scarification or other modifications of the grain surface may also affect positively the penetration of ions into the grain. One such surface modification technique could be the treatment of seeds or grains with cold or non-thermal plasma (CP). CP is generated by supplying energy to gas by heat, electricity, electromagnetic field at radio or microwave frequencies and thus increasing kinetic energy of electrons with consequent increase in the number of collisions between gas molecules and electrons, resulting in plasma discharge (18, 19). CP comprises ionized gas, including negatively and positively charged ions, free radicals, atoms, molecules, and in the case of low-pressure conditions UV and vacuum UV photons (20, 21). Depending on the gas used for the CP generation, reactive oxygen and/or nitrogen species (ROS and NOS) are also present (22). CP constituents interact with the surface of material and can decontaminate or even sterilize the surface (23). In the long run CP may also affect physiological properties, such as germination, yield, and/or even stress resistance in crops (21, 24, 25). In grains, CP treatment can make the surface more hydrophilic by functionalization of the surface molecules and/ or by mechanical etching of the surfaces (26–29). In oxygen-based CP treatment, the effect has been attributed to the functionalization of the surface by oxygen, and to oxidation of lipid molecules, largely present in the grain pericarp (26, 30–32), which leads to improved grain wettability and water uptake, and activates complex signaling pathways in seeds or grains (22, 29, 31, 33). The use of CP in agriculture is thus a promising environment-friendly technique that could help to reduce the use of pesticides and fertilizers and improve early-stage seedling vigor.

The aim of the study is to investigate whether Zn biofortification of sprouts can be enhanced by oxygen-based CP treatment of grains. By hypothesis, Zn uptake in CP-treated grains should increase, resulting in a higher Zn concentration in sprouts. The hypothesis was tested on common buckwheat, *Fagopyrum esculentum* Moench (Caryophyllales: Polygonaceae), a gluten-free crop, traditionally grown in Asia and Central and Eastern Europe. As buckwheat grain contains 57% of starch it is often used for the production of flour or is consumed as de-husked grains (groats), similarly as cereal grains. Consumption of buckwheat-based products has been linked to hypoglycemic, anti-cancer, and anti-inflammatory benefits (34, 35) making buckwheat an increasingly popular ingredient in healthy diets. Furthermore, buckwheat sprouts are health-promoting produce, rich with antioxidant and anti-inflammatory compounds (36–39); their element composition can be changed by the use of solution used to grow them (40).

## 2. Materials and methods

### 2.1. Plant material and experimental conditions

Grains of common buckwheat (*Fagopyrum esculentum* Moench) from the 2020 harvest was obtained from a local farm Rangus (Rangus mlinarstvo in trgovina, Dol. Vrhpolje d.o.o., Dolenje Vrhpolje 15, 8,310 Šentjernej, Slovenia). Between harvest and use in experiments, the grains were kept in closed glass jars in dry and dark place. Outline of all experiments is schematically presented in Supplementary material, Supplementary Figure A1.Germination tests were performed in Petri dishes (φ = 7 cm; 25 grains per Petri dish) lined with filter paper soaked in dH_2_O and kept in cardboard boxes (in the dark). Sprouts were grown on trays placed in sprouters (EasyGreen® MicroFarm System, Easy-Green Factory Inc., Nevada, United States), and watered with tap water by automatic misting for 30 min every 8 h. Petri dishes and sprouters were placed in a growth chamber where 16 h: 8 h day: night cycle was accompanied by 22°C and 19°C, respectively, at 60% humidity.

### 2.2. Grain-priming with different concentrations of ZnCl_2_

Different ZnCl_2_ concentrations were tested in three independent experiments to determine the optimum ZnCl_2_ concentration for subsequent experiments. 300 grains of common buckwheat were soaked for 16 h at room temperature in 100 mL of dH_2_O (Control) or in 100 mL of one of the following ZnCl_2_ aqueous solutions: 2.5 mM, 5 mM, 7.5 mM and 10 mM. After soaking period, grains were rinsed with tap water and 100 grains from Control and each of the ZnCl_2_ concentrations were used in germination test and the remaining 200 grains were used for sprout cultivation under the conditions described in “Plant material and experimental conditions”. Germinated grains were counted after five days and final germination rate G was calculated as:


(1) $$G=\frac{N_{g}\times 100\%}{N_{t}}$$

where N_t_ represents the total number of grains in the Petri dish, and N_g_ represents the number of germinated grains. Sprouts were harvested after 8 days: pericarps were removed from cotyledons, roots were discarded, and the shoots were weighed (fresh weight, FW), frozen in liquid nitrogen and freeze-dried (Alpha 2–4 Christ, Martin Christ Gefriertrocknungsanlagen GmbH, Osterode am Harz, Germany) for 3 days, after which the shoots were weighed again (dry weight, DW). The dried shoots were finely ground with pestle in a mortar. Around 300 mg of plant material was pressed into a pellet with a hydraulic press and analyzed with X-ray fluorescence (XRF) spectrometer (Peduzo T02, Jožef Stefan Institute, Ljubljana, Slovenia). The concentrations of phosphorus (P), sulfur (S), chlorine (Cl), potassium (K), Ca, manganese (Mn), Fe and Zn were measured as described in detail previously (41). On the remaining plant material the photosynthetic pigments (chlorophyll a and b, chlorophyll a + b and carotenoids) were determined following the procedure by Lichtenthaler (42).

### 2.3. Grain treatment with cold plasma

The CP treatment was conducted in a small-scale, inductively-coupled low-pressure oxygen plasma system in which plasma was powered by a radio-frequency (13.56 MHz) generator. Plasma discharge was established at 50 Pa working pressure, where the supply gas was oxygen of 99.99% purity. The direct (glow) plasma regime was used. To determine optimal power conditions of plasma, a test treatment for 5 s at three different power inputs of 75 W, 100 W and 200 W was done. Germination tests were performed on 100 grains from each power input and for the untreated (Control) sample, as described in “Plant material and experimental conditions”. The final germination rate (G) was calculated using the number of germinated grains on day ten.

For the Control samples and samples treated at 75 W, water contact angle (WCA) and water uptake was also determined. WCA was measured for pericarp of 10 randomly selected grains with Drop Shape Analyzer DSA 100E (KRÜSS GmbH, Hamburg, Germany) with 1 μL MiliQ water (StakPure, Niederahr, Germany) droplets, and with testing immediately and after one, two, three, six and twelve weeks. For water uptake determination 50 dry grains were weighed and placed onto one layer of filter paper soaked with 4 mL of dH_2_O in a Petri dish (as described in “Plant material and experimental conditions”). Weighing was repeated after 0.5, 1, 3, 6, and 24 h of grain exposure to water. Every time, any excess water was removed by a paper towel. In addition, grains pericarp of the control and CP-treated grains were imaged by a scanning electron microscope (SEM; Quanta 650 ESEM, Thermo Fischer, United States) without any surface coating.

### 2.4. Cold plasma grain pre-treatment and grain-priming

The effect of the CP pre-treatment at CP power of 75 W was studied in common buckwheat grains, which were primed for 16 h in dH_2_O (CP) or in 5 mM ZnCl_2_ solution (CP + Zn). Controls without CP pre-treatment and primed in dH_2_O (C) or in 5 mM ZnCl_2_ (+Zn) were examined for comparison, yielding four treatments.

Firstly, the distribution of elements in the grains was determined using micro-particle X-ray emission (micro-PIXE) on transverse cross-section made using a sharp stainless-steel platinum-coated razor blade. The cross-sections were frozen in liquid nitrogen and dried in a freeze-drier for 2 days. The dried sections were placed between two layers of Pioloform foil stretched over a custom-made aluminum frames (43). Five cross-sections per treatment were analyzed at the micro-PIXE set-up of the Jožef Stefan Institute, Slovenia (44, 45). X-ray fluorescence was detected with a segmented silicon drift detector (Rococo2 System, PNDetector GmbH, München, Germany). Quantitative element distribution maps were generated in GEOPIXE II software package (Ryan 2000) and the tissue specific concentrations of elements were extracted from the numerical matrices using Fiji program (46). Linear transects, one across the pericarp, aleurone, cotyledons and another one across the pericarp, aleurone and endosperm were obtained from a representative grain sample using PyMca software (47).

Secondly, α-amylase activity was measured in the grains, following user manual of CERALPHA kit (Megazyme, Wicklow, Ireland): after soaking the grains were rinsed with tap water, frozen in liquid nitrogen and freeze-dried as described above. Dried grains were finely ground with pestle in a mortar, and α-amylase was extracted from 250 mg of dried and homogenized material in a glass eprouvette. For each treatment three extractions of the samples (n = 3) were performed. Extraction of the samples was performed using 5 mL extraction buffer solution (pH 5.4). After thoroughly mixing the plant material with the extraction buffer (vortexing), the samples were incubated in a water bath for 20 min at 40°C and centrifuged at 1000 *g* for 10 min. For each sample, 100 μL of amylase reagent was transferred into 2 mL micro-centrifuge and pre-incubated in a water bath for 5 min at 40°C. One hundred μL of the sample was added to the amylase reagent, stirred and incubated in a water bath for further 10 min at 40°C. After the incubation, 1.5 mL of stopping reagent was added and the solution was vortexed. The samples were centrifuged at 1000 *g* for 10 min. The absorbance of the samples was read on the spectrometer (Schimatzu, UV-1880, Kyoto, Japan) at 400 nm. (A_400_) and α-amylase activity (CU g^−1^) was calculated using the following formula:


(2) $$\begin{array}{c} \alpha -\mathrm{amylase activity}=\frac{A400}{\mathrm{incubation time}}\times \frac{\mathrm{total volume in cell}}{\mathrm{volume of extract}}\\ \times 0.05525\times \frac{\mathrm{extraction volume}}{\mathrm{sample weight}}\times \mathrm{dilution}\times 4.1 \end{array}$$

Thirdly, three independent experiments with four technical replicates were conducted comprising germination tests and tests in sprouters as described in “Plant material and experimental conditions”. In germination tests, in addition to the final germination rate also the mean germination rate (MR) was calculated using the following formula (48):


$$\mathrm{MR}=\frac{1}{\frac{\sum N_{d}\times d}{N_{g}}}$$

where N_d_ represents the number of germinated grains on day d, and d represents the time (in days) from the start of the experiment. N_g_ represents the total number of germinated grains in the Petri dish. In experiments in sprouters, shoot biomass, the element composition and photosynthetic pigment content (as described in detail in “Grain-priming with different concentrations of ZnCl_2_”) were assessed, together with root biomass and element composition of the roots and pericarps.

### 2.5. Statistical analysis

The results were presented as mean ± standard error (SE) of the three independent experiments, each with three to four technical repetitions (n = 9 or 12), unless stated otherwise in figure and table captions. Treatment means were compared by one-way analysis of variance (ANOVA) with Holm-Sidak *post-hoc* test at *p* value <0.05 or when pairwise comparison was required, Student t-test at *p* value <0.05 was used. Statistical analysis and graphical visualization were carried out in SigmaPlot 12.0 software (Systat Software, San Jose, CA, United States).

## 3. Results and discussion

### 3.1. Optimizing conditions for grain-priming and cold plasma treatment of common buckwheat

**Figure 1:**
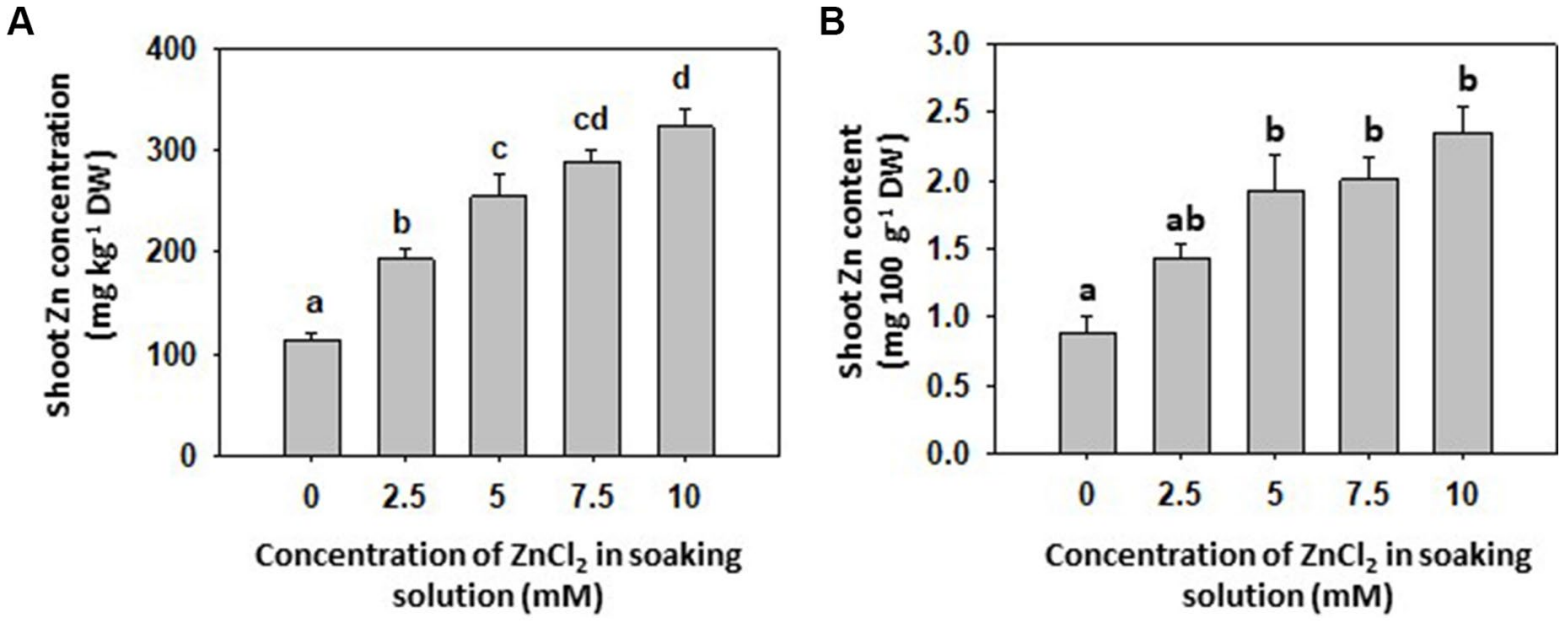
**(A)** Concentration of zinc (Zn) in shoots of 8-day-old sprouts of common buckwheat and **(B)** Zn content per one portion (100  g) of sprouts primed with increasing concentration of ZnCl_2_ (0, 2.5, 5, 7.5 and 10  mM) for 16 h at room temperature. Means and standard errors (*n* = 9) are shown. Different letters above columns (a, b, c, d) indicate significant differences (one-way ANOVA, Holm-Sidak *post-hoc* test at *p* < 0.05). DW, dry weight; FW, fresh weight.

Grain-priming with different ZnCl_2_ concentrations had no significant effect on final germination rate of grains, fresh and dry weight and photosynthetic pigments of common buckwheat sprout shoots (Table 1) confirming that selected ZnCl_2_ concentrations did not result in visible or measureable physiological and yield penalties. There was an increase in Zn concentration in shoots depending on the ZnCl_2_ concentrations used for priming (Figure 1). The maximum individual value 423 mg Zn kg^−1^ DW of shoot Zn concentration was measured in the 10 mM ZnCl_2_ treatment, and the minimum individual value 79 mg Zn kg^−1^ DW in the Control (0 mM ZnCl_2_), yielding 5.35-fold increase in Zn concentration. By contrast, grain-priming with ZnCl_2_ did not affect the concentrations of P, S, Cl, K, Ca, Mn and Fe in shoots (Table 1). By Zn soil fertilization, according to several studies, the concentration of Zn can be increased up to 700 mg kg^−1^ DW in shoots of some plant species, such as chickpea, different cabbages, turnip and spinach (1). Although there is no report on Zn biofortification attempts in buckwheat, a five-fold increase in Zn concentration after grain-priming can be considered a promising approach to improve Zn intake in human (or animal) diets. Taking into account that sprouts are consumed fresh, simple calculation reveals that a 100 g serving of fresh matter offers slightly more than 2 mg of Zn in sprouts originating from grains primed with 5 to 10 mM ZnCl_2_ (Figure 1B). Average requirement (AR) of Zn for adult men ranges between 7.5–12.7 mg per day and for adult women 6.2–10.2 mg per day (4) indicating the above portion of Zn-primed common buckwheat sprouts could account for around 20 and 25% AR, respectively. At least, Zn-primed common buckwheat (or any other, equally Zn-enriched) sprouts appear to be a good candidate to optimizing diets and/ or achieving improved Zn intake. As 5 mM ZnCl_2_ was the first treatment in which significantly more Zn was found in sprouts compared to Control, and as the content of Zn saturated with higher concentrations of ZnCl_2_, 5 mM ZnCl_2_ concentration was selected for further experiments.

**Table 1:** Final germination rate, biomass, concentration of photosynthetic pigments and of elements in shoots of 8-day-old sprouts of common buckwheat, whose grain was primed with increasing concentrations of ZnCl_2_ for 16  h at room temperature.

*Germination (%)*		
Final germination rate	56.4	±2.43
*Biomass (mg)*
Fresh weight	53.4	±0.84
Dry weight	7.38	±0.09
*Photosynthetic pigment (mg g^−1^ DW)*		
Chlorophyll a	18.7	±0.42
Chlorophyll b	17.9	±0.31
Chlorophyll a + b	36.5	±0.72
Carotenoids	1.55	±0.05
*Element concentration (mg kg^−1^ DW)*		
Phosphorus	2,039	±50.6
Sulphur	1,057	±23.7
Chlorine	1,234	±81.3
Potassium	3,764	±74.7
Calcium	6,507	±288
Manganese	47.2	±1.00
Iron	98.9	±1.84

As there was no statistically significant difference between samples, averages ± standard errors (*n* = 45) are shown. DW, dry weight.

**Figure 2:**
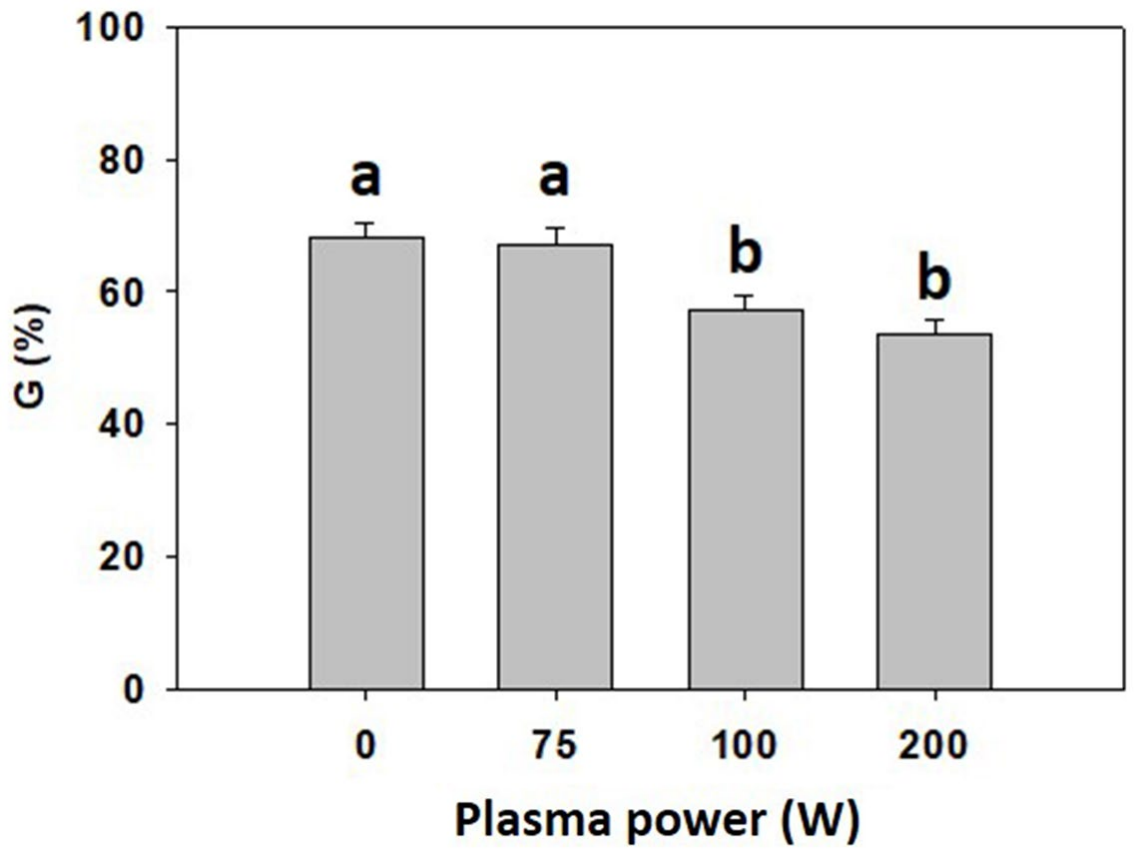
Final germination rate (G) of common buckwheat grain determined on the day 10 after treatment with cold plasma for 5  s at the power of 0  W, 75  W, 100  W, and 200  W. Shown are averages and standard errors (*n* = 12). Different letters above columns (a, b) indicate significant differences (one-way ANOVA, Holm-Sidak *post-hoc* test at *p* < 0.05).

To optimize CP power for 5 s-long treatment of common buckwheat grains, CP discharges of 75 W, 100 W and 200 W were tested along with untreated grains (0 W). A decrease in final germination rate was observed for grains exposed to plasma at 100 and 200 W compared to the untreated (0 W) and 75 W plasma treatment (Figure 2). Consequently, 75 W CP power was selected for the pre-treatment of the grains in subsequent experiments.

**Figure 3:**
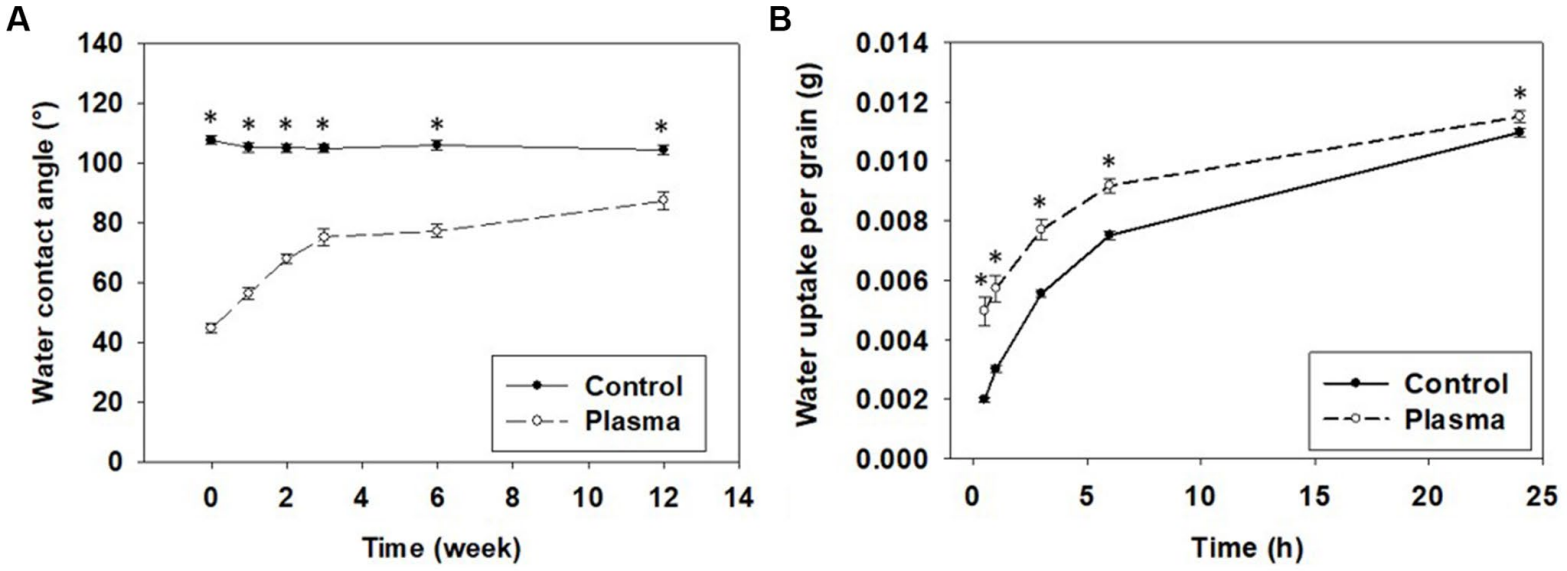
**(A)** Hydrophobic recovery of water contact angle and **(B)** water uptake per grain of non-treated (Control) and cold-plasma-treated (Plasma) common buckwheat grain. Shown are means and standard errors (*n* = 30). The asterisks indicate significant difference between treatments (Student *t*-test at *p* < 0.05).

The selected 75 W power of CP treatment was further examined for its effects on the common buckwheat grain pericarp, in particular the hydrophilic properties of the grain surface and the amount of water uptake. Hydrophilic property was determined through WCA, which was conducted immediately after the CP treatment, when a significantly smaller WCA value was measured for the CP-treated grains (44.6°) than for Control grain (107.7°) as shown in Figure 3A. The higher hydrophilicity of the pericarp surface is mainly due to functionalization of surface with oxygen functional groups and is in line with similar reports for different plant species (22, 26, 29, 31, 33). It can be attributed to the oxidation of lipids on the pericarp surface occurring due to the oxygen CP being used as observed for wheat grain (30). A protective waxy layer of lipids covering the surface of the grain, is oxidized by the ROS in the CP (31, 32). These effects, however, were not visible on the SEM micrographs (Supplementary material, Supplementary Figure B1) indicating that the morphological changes taking place are either finer than the resolution of the SEM or are of only chemical nature.

With measurements repeated after one, two, three, six and twelve weeks, the WCA gradually increased for the CP-treated grains up to the third week and did not change much further on (Figure 3A). At all time-points the WCA was re-evaluated and it remained lower for the CP treated grains compared to Control grains, whose WCA was practically constant with time. This observation suggests that the improved hydrophilicity, gained as a result of the CP treatment, was not stable with time. The so-called aging after plasma treatment or hydrophobic recovery is a well-known effect observed for plasma treated polymers, where due to energetically more stable state, the polymer surface restores (at least partially) hydrophobic properties over time (49, 50). However, it has been thought that hydrophobic recovery is not present after CP treatment of grains and has not been reported for a long time (26, 51). The first report of the phenomenon has been published recently on CP-treated Bambara groundnut grains (52). The authors suggested that the hydrophobic recovery was not reported previously due to a much slower recovery rate of hydrophobicity in grains compared to polymers (52).

The changed hydrophilic properties of the grain surface can improve water uptake of grains (29, 53–55). In accordance, CP-treated common buckwheat grains showed a significantly higher water uptake than the Control grains (Figure 3B). Our results thus confirm that CP treatment of common buckwheat grains caused an increase in hydrophilicity of the grain pericarp, resulting in higher wettability of the grains, leading to improved water uptake, as reported in other studies for different plant species (53, 56). The improved water uptake by grains after CP treatment may be advantageous in grain-priming as it may shorten the soaking time. Further evaluations will be required to confirm this hypothesis.

### 3.2. Effects of cold plasma pre-treatment and grain-priming on element distribution in the grains and partitioning of elements in common buckwheat sprouts

**Figure 4:**
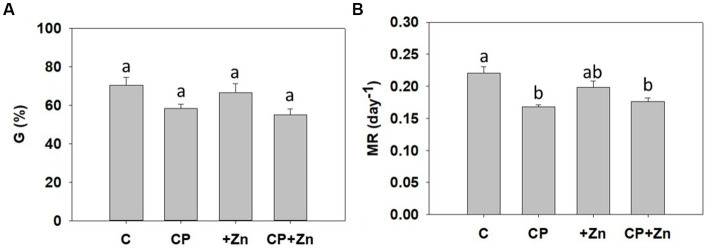
**(A)** Final germination rate (G) and **(B)** mean germination rate (MR) of common buckwheat grain on day ten. The grain was not-pre-treated (C) or pre-treated with cold-plasma for 5  s at 75  W (CP), followed by soaking in dH_2_O (C and CP) or in 5  mM ZnCl_2_ (+Zn and CP + Zn) for 16  h at room temperature. Means and standard errors (*n* = 12) are shown. Different letters above columns (a, b) indicate significant differences (one-way ANOVA, Holm-Sidak *post-hoc* test at *p* < 0.05).

Common buckwheat grains were pre-treated with CP for 5 s at 75 W and/or primed by soaking in 5 mM ZnCl_2_ for 16 h at room temperature. There were no significant differences in the final germination rate between different treatments (Figure 4A). Likewise, treatments did not affect α-amylase activity or fresh and dry biomass, with respective statistical values 0.45 ± 0.06 IU, 54.2 ± 1.03 mg and 7.72 ± 0.136 mg. Important enzymes in grain germination, α-amylases, release the starch reserves in the grains upon imbibition and providing nutrients for the growth and development of embryo during germination (57). As there was no effect of CP treatment on the activity of this important germination-related enzyme, it appears that CP pre-treatment did not affect the metabolism in the early stages of germination. The only significant effect was observed in the mean germination rate (i.e., the index of germination speed): grains from CP and CP + Zn treatments germinated more slowly than the Control (Figure 4B). Regardless of the decrease in germination speed, which suggests early stress for the grains, the final germination rate was not affected and the biomass production in sprouts of all treatments was unperturbed, as demonstrated already for tomato seeds (58).

**Figure 5:**
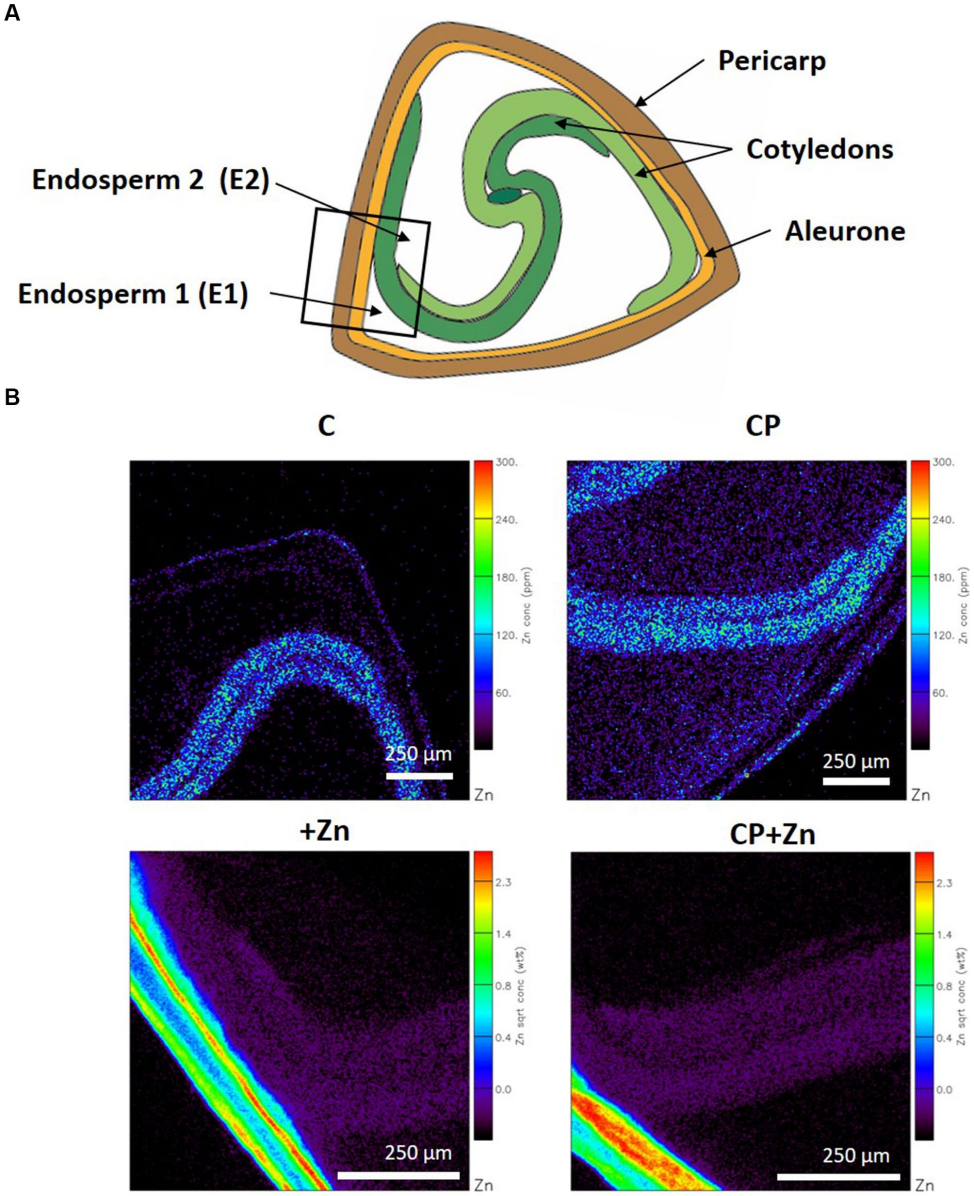
**(A)** Common buckwheat grain cross-section with the square indicating the area of element mapping, and **(B)** representative distribution maps of zinc (Zn). Grain was not-pre-treated (C) or was pre-treated with cold plasma for 5  s at 75  W (CP), followed by soaking in dH_2_O (C and CP) or in 5  mM ZnCl_2_ (+Zn and CP + Zn) for 16  h at room temperature. Color legends indicate concentration in % dry weight.

To evaluate effects of CP pre-treatment and grain-priming on Zn diffusion into the grains, the detailed tissue-specific element composition of the grains was determined. Multielement localization technique, micro-PIXE, was used on cross-sections of the grains from the four treatments. In a cross-section of common buckwheat grain pericarp, aleurone, cotyledons, and endosperm were recognized; for endosperm two locations were distinguished: the first (E1) was between the aleurone and cotyledon and the second (E2) between the cotyledons (Figure 5A). The two locations of endosperm were investigated to assess the level of restriction, both physical and physiological, that outer layers present in the passage of Zn from outside into the center of the grains. Since for E2, the cotyledons represented an additional barrier, less Zn was expected there.

In each grain, selected areas comprising all five tissues were mapped as indicated in Figure 5A. Zn distribution maps clearly indicate that in C and CP grains the highest concentration of Zn was in cotyledons, while in +Zn and CP + Zn grains, pericarp contained more Zn than any other tissue (Figure 5B).

**Figure 6:**
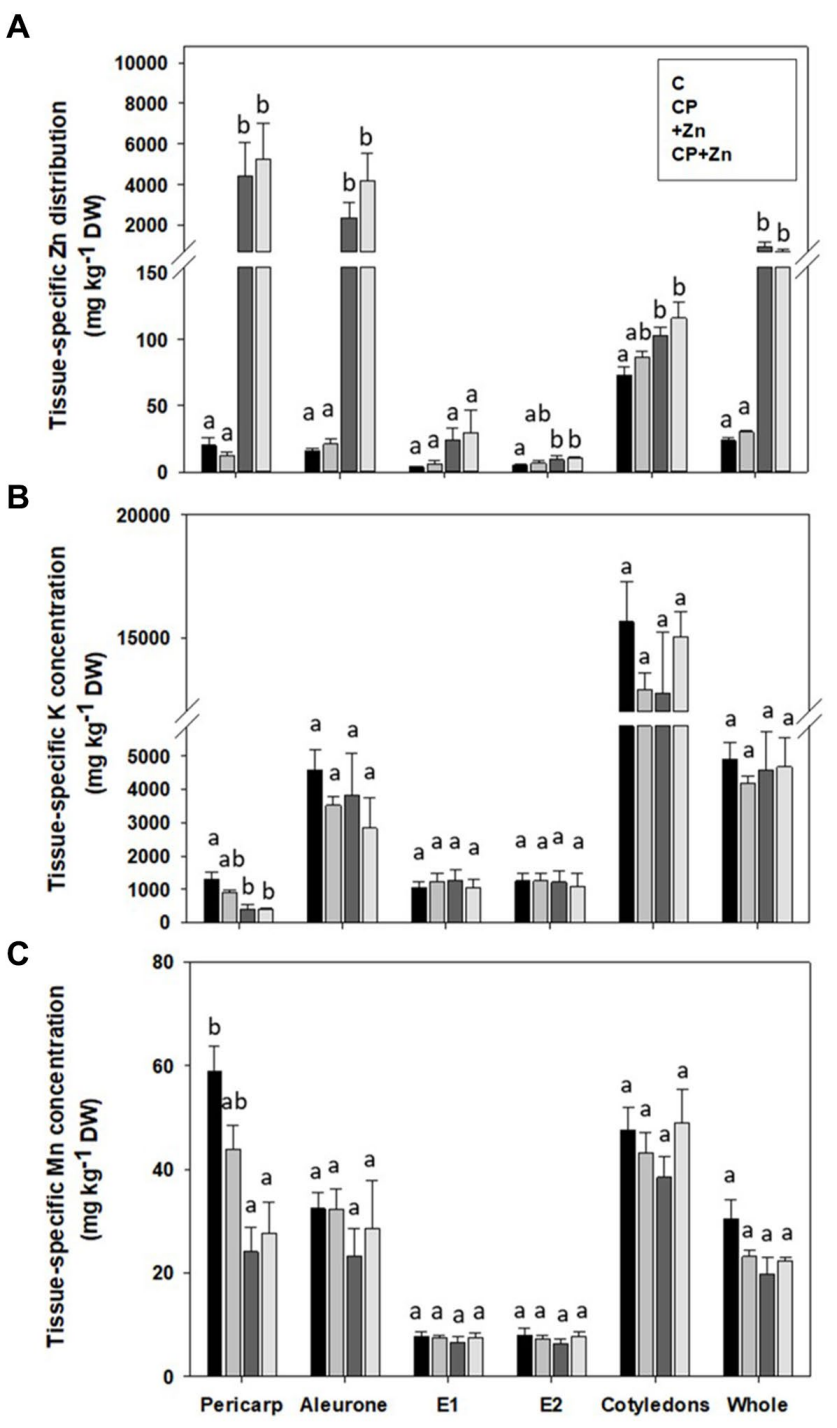
Tissue-specific concentration of **(A)** zinc (Zn), **(B)** potassium (K), and **(C)** manganese (Mn) in common buckwheat grain. Grain was not-pretreated (C) or pre-treated with cold-plasma for 5 s at 75 W (CP), followed by soaking in dH_2_O (C and CP) or in 5  mM ZnCl_2_ (+Zn andCP + Zn) for 16  h at room temperature. Concentrations were determined for the entire cross-section (whole) and the followinggrain tissues: pericarp, aleurone, endosperm between aleurone andcotyledon (E1), endosperm after the cotyledons (E2) and cotyledons.Shown are means and standard errors (*n* = 5). Different letters above columns (a, b) indicate significant differences between treatmentsfor each tissue separately (one-way ANOVA, Holm-Sidak post-hoctest at *p *< 0.05). DW, dry weight.

Because micro-PIXE is a quantitative technique, tissue-specific concentrations of detected elements (for the set-up used in the study these elements were P, S, Cl, K, Ca, Mn, Fe, and Zn) can be determined. Entire grain cross-section, pericarp and aleurone of Zn-primed grains had higher Zn concentration than not-primed grains; at E2 the concentration was higher in Zn-primed grains (+Zn and CP + Zn) than in control grains (C and CP) (Figure 6A). There was no effect of CP pre-treatment on Zn concentration in any of the grain tissues.

Of the other elements detected, only K and Mn concentrations were significantly affected by the treatments +Zn and CP + Zn, and only in pericarp: Zn-primed grains contained less K and Mn than Control grains (Figures 6B,C). Tissue-specific concentration of other elements are presented in Supplementary Figure B1.

**Figure 7:**
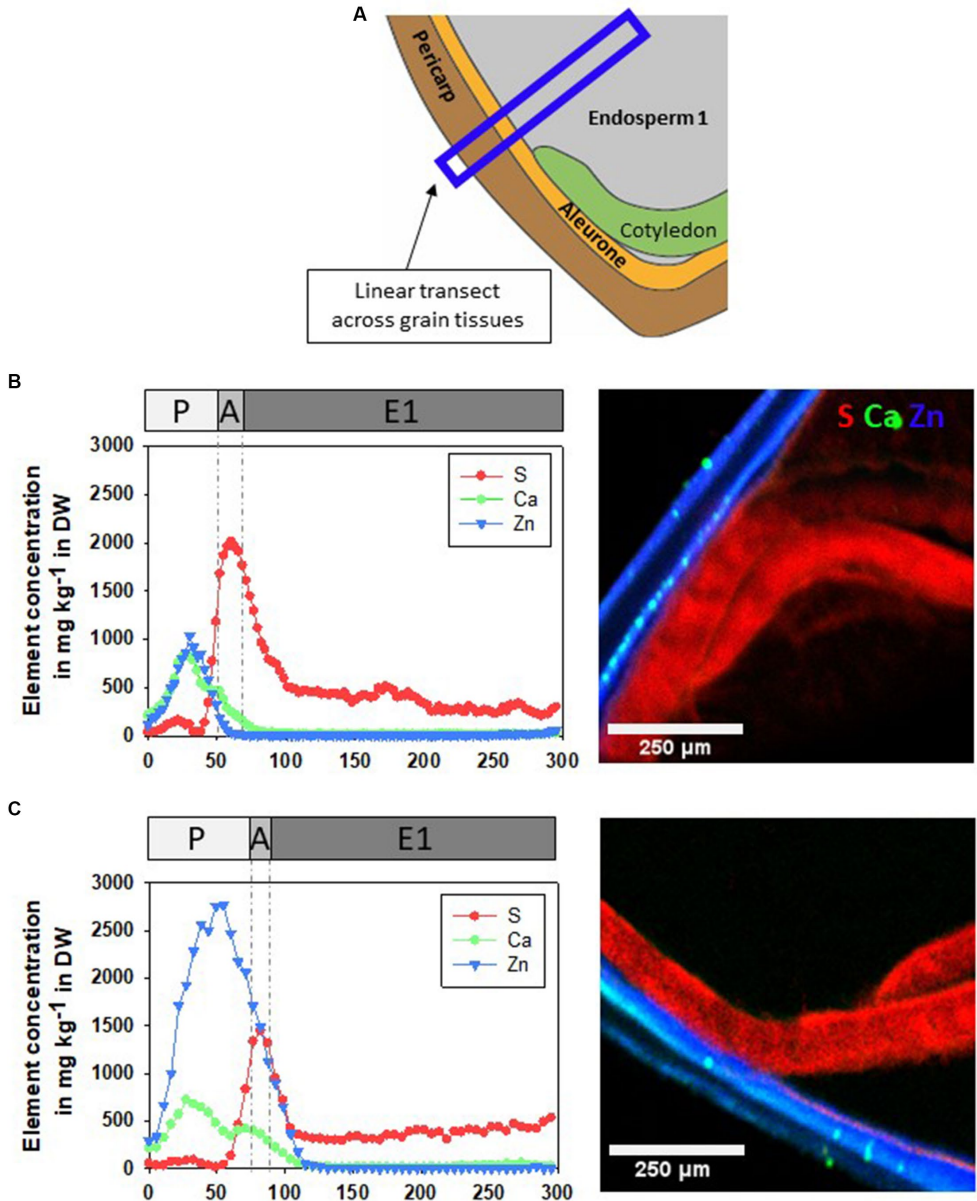
**(A)** A schematic representation of a position of linear transect across pericarp (P), aleurone (A), cotyledons (C) and endosperm (E1) in a cross-section of +Zn and CP + Zn grains. Concentration of sulfur (S), calcium (Ca), and zinc (Zn) in linear transect and co-localisation of S (in red), Ca (in green) and Zn (in blue) in the representative cross section. Grains were soaked in 5 mM ZnCl_2_ for 16 h at room temperature after **(B)** not being pre-treated (+Zn) or **(C)** were pre-treated with cold plasma for 5 s at 75 W (CP+Zn). DW, dry weight.

Because there was an insignificant trend of higher concentration of Zn in pericarp and aleurone of CP-pre-treated and Zn-primed grains compared to Zn-primed grains only, linear transects across grain tissues (pericarp, aleurone and E1) were inspected (Figure 7A). To better visualize grain tissues depicted, the colocalization of Ca, S and Zn was generated. The highest concentration of Ca was found in pericarp and of S (a proxy for proteins) in cotyledons and in aleurone, in line with previous reports (45, 59, 60). In the linear transect theZn-primed grains (Figure 7B) had more Zn across the pericarp (aligned with Capeak) compared to CP+Zn grains (Figure 7C), but no significant differencesbetween the treatments were found deeper in the grains (aleurone,cotyledons and endosperm 1).

**Figure 8:**
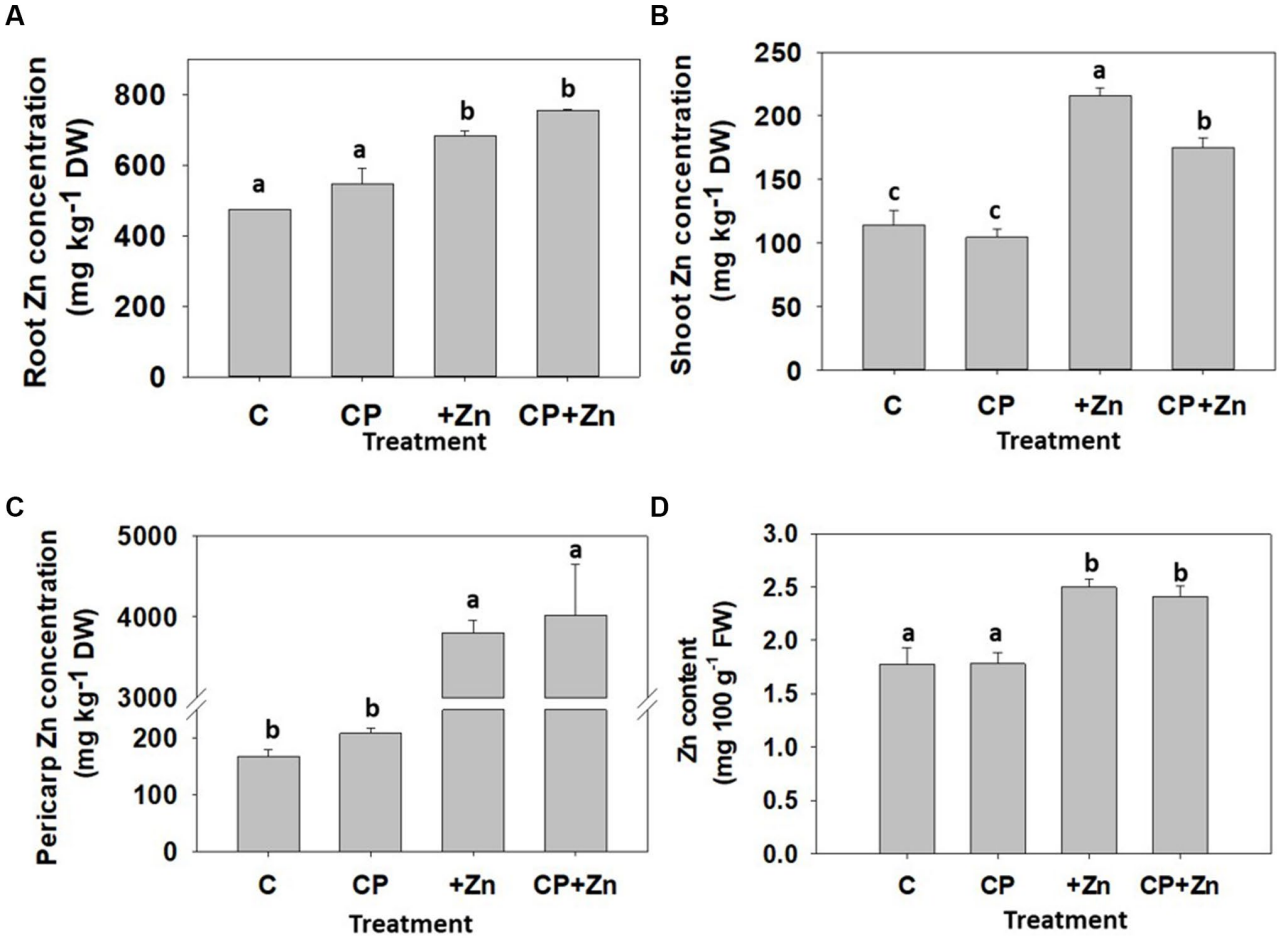
Concentration of zinc (Zn) in **(A)** roots, **(B)** shoots, **(C)** pericarps and **(D)** Zn content in whole sprouts per 100  g of fresh weight of 8-day-old common buckw-heat sprouts, whose grain was not-pre-treated (C) or pre-treated with cold plasma for 5  s at 75  W (CP), followed by soaking in dH_2_O (C and CP) or in 5 mM ZnCl_2_ (+Zn and CP + Zn) for 16  h at room temperature. Shown are means and standard errors (*n* = 9). Different letters above columns (a, b, c) indicate significant differences (one-way ANOVA, Holm-Sidak *post-hoc* test at *p* < 0.05). DW, dry weight; FW, fresh weight.

The effect of CP pre-treatment followed by Zn priming of the common buckwheat grains on element composition and partitioning of 8-day-old sprouts was also examined. To capture the element partitioning, the sprouts were separated into roots and shoots. The pericarps, which shed off the cotyledons during sprout development, were also collected. In sprouts from Zn-primed grains, Zn concentration was the highest in pericarp followed by the roots and the shoots (Figure 8). The concentration of Zn in all parts was significantly higher in sprouts from Zn-primed grains than in non-primed grains, particularly for pericarps, where 20.8-fold difference was found (Figure 8C), while for shoots (Figure 8B) and roots (Figure 8A) a 2-fold and 1.4-fold difference, respectively, was found. A 100 g portion including fresh roots and shoots and excluding inedible pericarps would result in 2.45 mg of Zn (Figure 8D) which represents around 24.5 and 35% of AR for adult men and women, respectively. Considering consumption of whole sprouts, i.e., including fresh roots and shoots in a portion, the negative effect of CP observed for shoot Zn concentration on dry weight basis, is no longer found (Figure 8D). Presumably this is a result of slightly (but not significantly) higher Zn concentration in roots of CP pre-treated grains, which warrants further investigation because embryonic axis (embryo proper) is positioned on the pointed (distal) part of the common buckwheat grains (45), therefore may have been affected by the CP.

CP pre-treatment did not significantly affect the Zn concentration in roots and pericarps, but it lowered Zn concentration in shoots (Figure 8) in comparison to sprouts from Zn-primed grains. The concentration of Zn in pericarps still attached to the grains, right after soaking in the ZnCl_2_, was higher (1.24-fold) compared to the pericarps removed from 8-day-old sprouts (Figure 6), which indicates that Zn is bound lightly to the pericarp and leaches off during 8-day long sprout development, becoming available for uptake by roots.

Among other elements detected, pericarp contained the lowest concentrations of P, S, Cl and Ca, the most Ca and Fe and similar concentrations of Mn as roots and shoots (Table 2). The concentration of S, Cl, K and Fe were higher in roots, while concentrations of P and Ca were of similar range in roots and shoots (Table 2). Interestingly, although there was no treatment effect on Cl concentration in pericarp immediately after soaking in ZnCl_2_ (Supplementary material, Supplementary Figure C1), there was an increase in Cl concentration in pericarp from 8-day-old sprouts (Table 2) from primed grains compared to non-primed grains, with the highest Cl concentration in pericarp found in the CP + Zn treatment (Table 2). It can be speculated that this observation is due to CP pre-treatment changing the grains surface favorably for the stronger adsorption of Cl^−^ during grain-priming. Consequently, there was less Cl removed from CP + Zn pericarps than from +Zn pericarps. In general Cl^−^ has a small binding affinity (61) presumably due to the negatively charged cell walls and pericarps. Similar effects were seen for P and S concentrations in pericarps (Table 2), but in contrast to Cl these two elements were not added to the solution for grain soaking. The anionic P and S may be more readily adsorbed to the pericarps; however, their origin remains unknown. Improved adhesion of organic surfaces exposed to CP have been already reported supporting our speculation (62, 63).

**Table 2:** Concentration of elements in roots, shoots and pericarps (in mg kg^−1^ of dry weight) of 8-day-old sprouts of common buckwheat without plasma pre-treatment (C) or cold-plasma pre-treated for 5  s at 75  W (CP), followed by soaking in dH_2_O (C and CP) or in 5  mM ZnCl_2_ (+Zn and CP + Zn) for 16  h at room temperature.

Treatment	Phosphorus	Sulphur	Chlorine	Potassium	Calcium	Manganese	Iron
*Roots*							
Average across treatments	1,449 ± 49	1,055±42	1,167±58	12,008±347	4,221±86	29±3	120±7
*Shoots*							
C	1,835±126**a**	937±23	708±51	3,706± 68	4,727±158	50±1	101±1
CP	1,464±131**b**						
+Zn	1,625±70**ab**						
CP + Zn	1824±104**a**						
*Pericarp*							
C	435±15**b**	189±22**c**	103±12**c**	166±8	6,449±20	58±3**a**	246±17
CP	444±28**b**	142±8**c**	124±19**c**			53±3**a**	
+Zn	820±129**a**	230±25**b**	320±15**b**			38±8**b**	
CP + Zn	700±126**b**	415±129**a**	418±166**a**			39±4**b**	

Different letters (a, b) indicate significant differences between treatments for each element separately (one-way ANOVA, Holm-Sidak *post-hoc* test at *p* < 0.05). Shown are means and standard errors (*n* = 3 or, when there was no significant difference between treatments, *n* = 20).

Lower concentrations of Mn in pericarps immediately after soaking (micro-PIXE results; Figure 6) and in 8-day-old sprouts (XRF bulk results; Table 2) can be linked to the priming process, during which Zn may have been displacing Mn from the grain surface. Interestingly such displacement did not take place with Fe and no treatment effects on Fe concentrations was observed (Table 2), possibly due to a stronger Fe binding to the pericarp than Mn. Clearly, further in-depth studies are needed to resolve the open questions of interaction of other essential elements with high Zn concentration resulting from grain-priming with ZnCl_2_.

## 4. Conclusion

It was shown that it is possible to increase Zn concentration in common buckwheat sprouts by grain-priming in ZnCl_2_. Ideally, both roots and shoots should be consumed, together providing 24.5 and 35% of Zn recommended daily allowance for men and women, respectively. Selected cold plasma treatment (5 s-long direct glow at 75 W) of common buckwheat grains resulted in better wettability and water contact angle. It lowered the speed of germination, but the final germination rate of grains and α-amylase activity did not decrease compared to control treatments. Combining cold plasma pre-treatment with grain-priming did not increase Zn concentration in grain tissues or in sprouts more than did the grain-priming alone, but it resulted in interactions with P, S, Cl and Mn in the pericarp, which were presumably due to chemical modifications of the surfaces by cold plasma. The applicability of the cold plasma pre-treatment followed by grain priming to biofortify sprouts with Zn (or other essential elements often lacking in diets) needs to be further investigated to optimize the experimental conditions of cold plasma pre-treatment and soaking of grains in element-rich solution(s). Another consideration wouldbe to apply the combination of these two technologies for other edible sprouts, particularly those with edible grain coats and/ or husk.

## Data availability statement

The raw data supporting the conclusions of this article will be made available by the authors, without undue reservation.

## Author contributions

PS, PP, PV, KV-M, and IJ contributed to conception and design of the study. PS, LR, MK, and PP organized the database. PS, PP, LR, and KV-M performed the statistical analysis. Visualization was done by MK, PV, and IJ. PS, LR, and PP wrote the first draft of the manuscript. IJ, KV-M, MK, and PV wrote sections of the manuscript. All authors contributed to manuscript revision, read, and approved the submitted version.

## Funding

The work was funded by the Slovenian Research Agency (ARRS) through program groups [P2-0082, P1-0212, and P1-0112], ARRS young research grant [PS], ARRS projects [J4-3091 and J1-3014] and project supported by ARRS and Ministry of agriculture, forestry and food [CRP V4-2001], and the infrastructural center “Centre for Electron Microscopy and Microanalysis” of the Jožef Stefan Institute.

## Conflict of interest

The authors declare that the research was conducted in the absence of any commercial or financial relationships that could be construed as a potential conflict of interest.

## Publisher’s note

All claims expressed in this article are solely those of the authors and do not necessarily represent those of their affiliated organizations, or those of the publisher, the editors and the reviewers. Any product that may be evaluated in this article, or claim that may be made by its manufacturer, is not guaranteed or endorsed by the publisher.
