# Supplementary material for: Exploring the potential of cold plasma treatment followed by zinc-priming for biofortification of buckwheat sprouts
Source: Front Nutr. 2023 May 5;10:1151101. doi: 10.3389/fnut.2023.1151101 (PMC10196170; doi:10.3389/fnut.2023.1151101)
Supplement: Supplementary file 1 [file Data_Sheet_1.docx]

Supplementary Material

Exploring the potential of cold plasma treatment followed by zinc-priming for biofortification of buckwheat sprouts

Pia Starič^1, 2^, Lucija Remic^2^, Katarina Vogel-Mikuš^1,2^, Ita Junkar^1^, Primož Vavpetič^1^, Mitja Kelemen^1^, Paula Pongrac^1,2*^

*** Correspondence:** Paula Pongrac: [paula.pongrac@bf.uni-lj.si](mailto:paula.pongrac@bf.uni-lj.si)

**Figure A1.** Workflow of the experiments.

**Figure B1.** Scanning electron micrographs of common buckwheat grain pericarp. Grain was either untreated (C) or cold-plasma pre-treated for 5 s at 75 W (CP).

**Figure C1.** Tissue-specific concentration of phosphorus (P), sulphur (S), chlorine (Cl), calcium (Ca) and iron (Fe) in common buckwheat grain. Grains were either untreated (C) or cold-plasma pre-treated for 5 s at 75 W (CP), followed by soaking in dH_2_O water or in 5 mM ZnCl_2_ (+Zn and CP+Zn) for 16 hours at room temperature. Concentrations were determined for the entire cross-section and the following grain tissues: pericarp, aleurone, endosperm between aleurone and cotyledon (E1), endosperm after the cotyledons (E2) and cotyledons. Shown are means and standard errors (n=5); DW, dry weight.


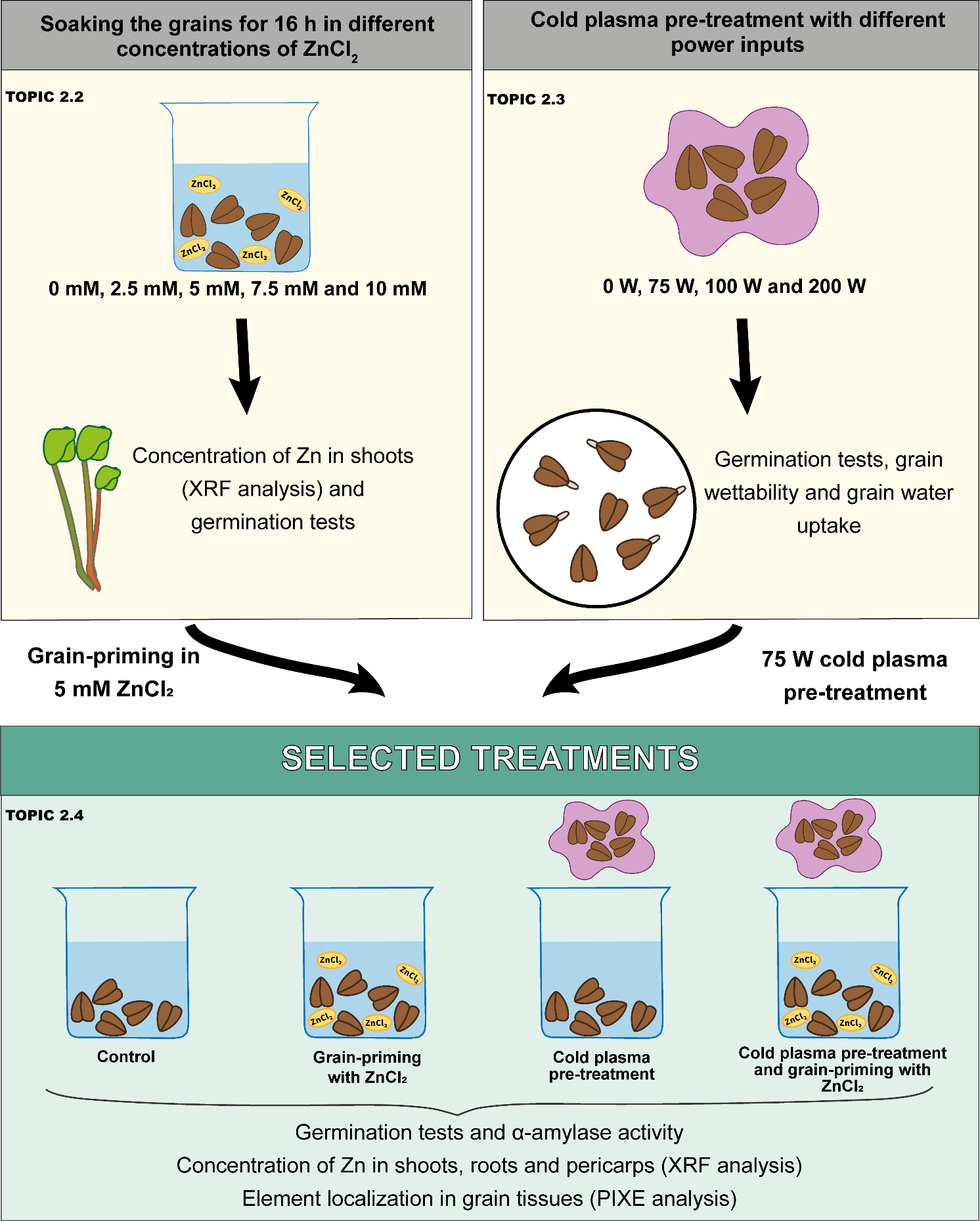


**Figure A1.** Workflow of the experiments.


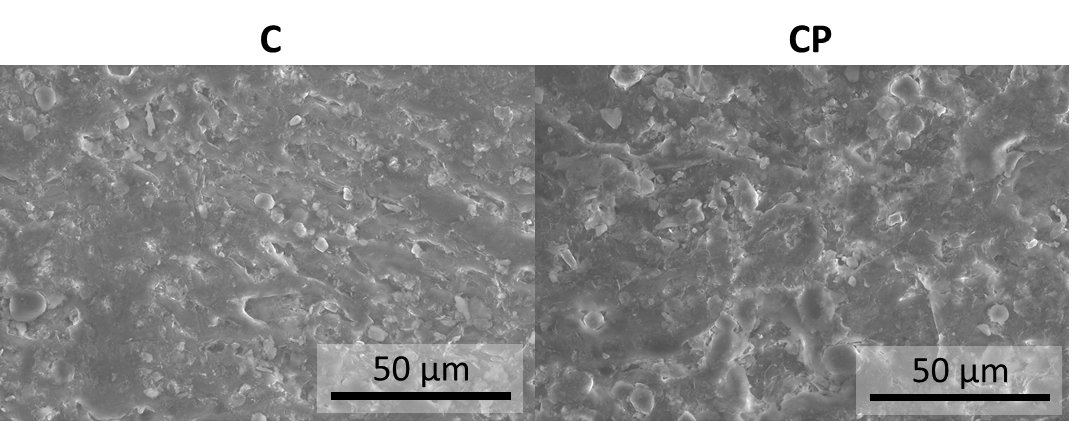


**Figure B1.** Scanning electron micrographs of common buckwheat grain pericarp. Grain was either untreated (C) or cold-plasma pre-treated for 5 s at 75 W (CP).


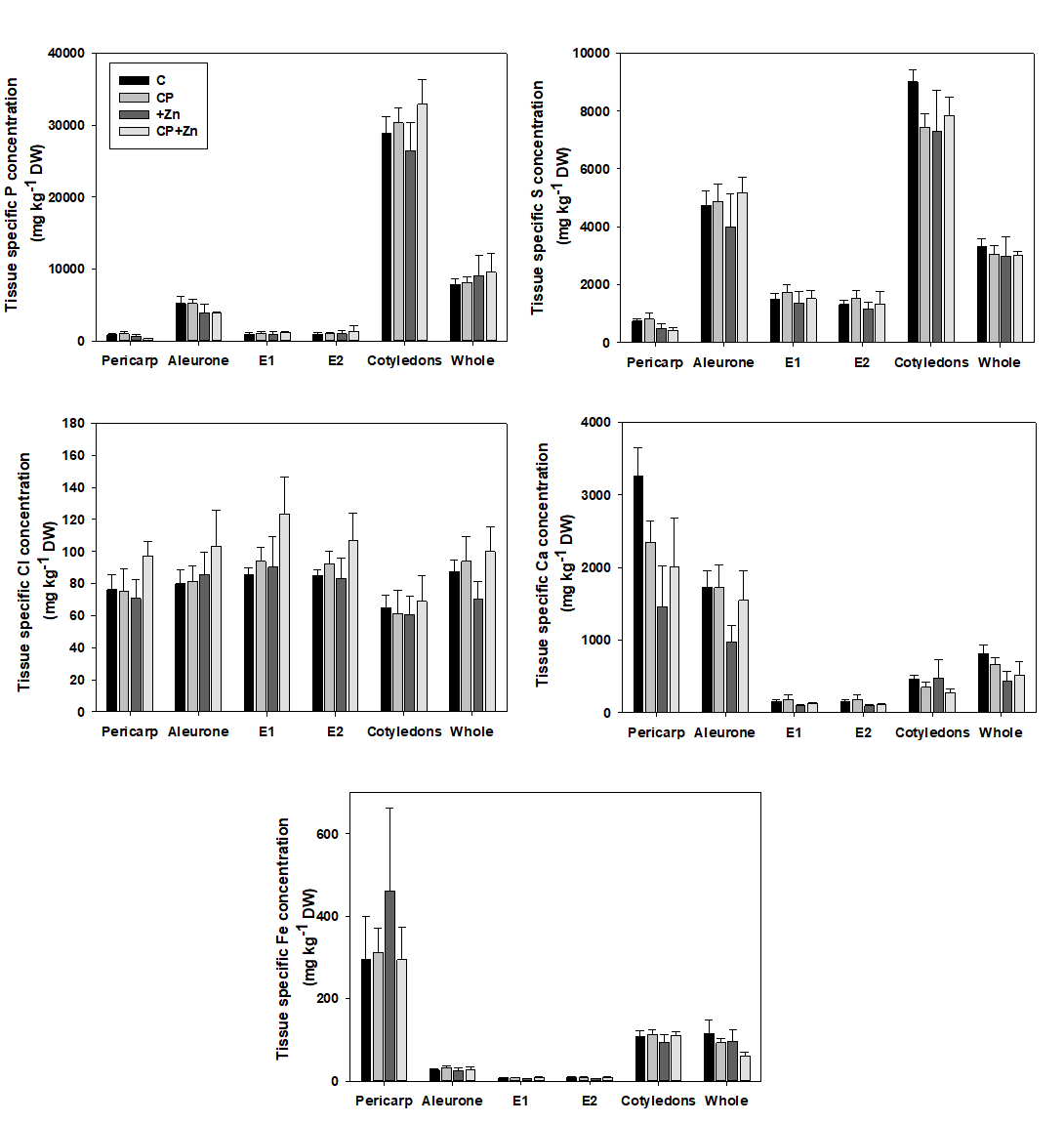


**Figure C1.** Tissue-specific concentration of phosphorus (P), sulphur (S), chlorine (Cl), calcium (Ca) and iron (Fe) in common buckwheat grain. Grains were either untreated (C) or cold-plasma pre-treated for 5 s at 75 W (CP), followed by soaking in dH_2_O water or in 5 mM ZnCl_2_ (+Zn and CP+Zn) for 16 hours at room temperature. Concentrations were determined for the entire cross-section and the following grain tissues: pericarp, aleurone, endosperm between aleurone and cotyledon (E1), endosperm after the cotyledons (E2) and cotyledons. Shown are means and standard errors (n=5); DW, dry weight.

**
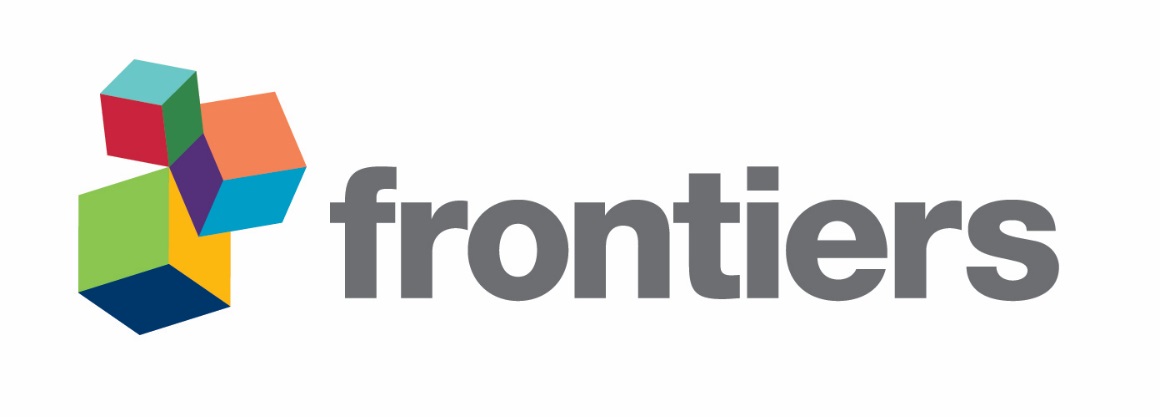
**
